# Effects of different plasma expanders on rats subjected to severe acute normovolemic hemodilution

**DOI:** 10.1186/s40779-020-00282-4

**Published:** 2020-11-11

**Authors:** Guo-Xing You, Bing-Ting Li, Zhen Wang, Quan Wang, Ying Wang, Jing-Xiang Zhao, Lian Zhao, Hong Zhou

**Affiliations:** Institute of Health Service and Transfusion Medicine, Bejing, 100850 China

**Keywords:** Acute normovolemic hemodilution, Plasma expander, Acid-base, Lactate, Blood gas, Physiological parameters

## Abstract

**Background:**

Plasma expanders are widely used for acute normovolemic hemodilution (ANH). However, existing studies have not focused on large-volume infusion with colloidal plasma expanders, and there is a lack of studies that compare the effects of different plasma expanders.

**Methods:**

The viscosity, hydrodynamic radius (R_h_) and colloid osmotic pressure (COP) of plasma expanders were determined by a cone-plate viscometer, Zetasizer and cut-off membrane, respectively. Sixty male rats were randomized into five groups with Gelofusine (Gel), Hydroxyethyl Starch 200/0.5 (HES200), Hydroxyethyl Starch 130/0.4 (HES130), Hydroxyethyl Starch 40 (HES40), and Dextran40 (Dex40), with 12 rats used in each group to build the ANH model. ANH was performed by the withdrawal of blood and simultaneous infusion of plasma expanders. Acid-base, lactate, blood gas and physiological parameters were detected.

**Results:**

Gel had a lower intrinsic viscosity than HES200 and HES130 (*P <* 0.01), but at a low shear rate in a mixture of colloids, red cells and plasma, Gel had a higher viscosity (*P* < 0.05 or *P* < 0.01, respectively). For hydroxyethyl starch plasma expanders, the COP at a certain concentration decreases from 11.1 mmHg to 6.1 mmHg with the increase of R_h_ from 10.7 nm to 20.2 nm. A severe ANH model, with the hematocrit of 40% of the baseline level, was established and accompanied by disturbances in acid-base, lactate and blood gas parameters. At the end of ANH and 60 min afterward, the Dex40 group showed a worse outcome in maintaining the acid-base balance and systemic oxygenation compared to the other groups. The systolic blood pressure (SBP), diastolic blood pressure (DBP), and mean arterial pressure (MAP) decreased significantly in all groups at the end of ANH. The DBP and MAP in the Dex40 group further decreased 60 min after the end of ANH. During the process of ANH, the Dex40 group showed a drop and recovery in SBP, DBP and MAP. The DBP and MAP in the HES200 group were significantly higher than those in the other groups at some time points (*P* < 0.05 or *P* < 0.01).

**Conclusion:**

Gel had a low intrinsic viscosity but may increase the whole blood viscosity at low shear rates. R_h_ and COP showed a strong correlation among hydroxyethyl starch plasma expanders. Dex40 showed a worse outcome in maintaining the acid-base balance and systemic oxygenation compared to the other plasma expanders. During the process of ANH, Dex40 displayed a V-shaped recovery pattern for blood pressure, and HES200 had the advantage in sustaining the DBP and MAP at some time points.

## Background

Acute normovolemic hemodilution (ANH) is performed ahead of a procedure with a high risk of blood loss [1]. During ANH, whole blood from the patient is removed and replaced with a mixture of crystalloids and colloids to maintain the blood volume [1, 2]. ANH is widely applied for patients coming through cardiac surgery, particularly for those who refuse transfusion for religious or other reasons [3]. Moreover, ANH shows great prospects in patients coming through major hepatic surgery and other procedures associated with moderate-to-high blood loss (at least 1000 ml) [4]. ANH is also an effective and safe way to reduce the need for perioperative transfusion in pediatric patients receiving high blood-loss surgeries [5]. A meta-analysis has demonstrated that patients undergoing ANH have a clinically related reduction in red blood cell transfusions and a decreased incidence of transfusions with allogeneic blood [6]. ANH is regarded as an important clinical strategy for decreasing the use of allogeneic blood [7] and has been approved as a standard method of intraoperative blood conservation by the American Society of Anesthesiologists [3, 7].

The crystalloids and colloids used in ANH include balanced salt solutions, albumin solutions, and artificial plasma expanders. Plasma expanders are used commonly in mainland China and include succinylated gelatin injection (Gelofusine, Gel), Dextran 40 sodium chloride injection (Dex40), 200/0.5 hydroxyethyl starch and sodium chloride injection (Hydroxyethyl Starch 200/0.5, HES200), 130/0.4 hydroxyethyl starch and sodium chloride injection (Hydroxyethyl Starch 130/0.4, HES130) and hydroxyethyl starch 40 sodium chloride injection (Hydroxyethyl Starch 40, HES40).

Hydroxyethyl starch (HES) solutions, which is also commonly applied in shock treatments to increase the plasma volume [8], is a colloid solution classified by the molecular weight and the degree of substitution [9]. Gelatins are semisynthetic colloids obtained from the breakdown of collagen [9]. The use of HES130 in patients undergoing acute hemodilution reportedly results in better microvascular reactivity compared to the use of Gel [10]. Dextrans are polysaccharides that are available in multiple molecular weights [9], among which Dextran 70, Dextran 40 and Dextran 20 are widely used clinically. Animal studies have concluded that dextrans are effective plasma expanders that can decrease edema formation compared with crystalloids [11].

In recent years, the safety of using the above mentioned colloids intraoperatively has been extensively debated [12]. Large randomized controlled trials [13] have reported that the use of HES130 is associated with an increased risk of renal dysfunction in patients requiring renal replacement therapy and a higher mortality rate in patients with severe sepsis who receive 6% HES130 [14]. The US Food and Drug Administration (FDA) has recommended that HES should not be applied in critically ill patients [9].

Despite these concerns, plasma expanders are still appropriate in certain situations. One study [15] reported that a patient, whose blood type was B and Rh-negative, successfully underwent extreme hemodilution with HES130, and another study [8] reported that HES is effective in improving acidosis in patients with aluminum phosphide poisoning. Furthermore, the Coordination Group for Mutual Recognition and Decentralized Procedures-Human, which is a medicinal regulatory body representing the European Union Member States, decided that HES should be used for infusion in clinical practice, provided that additional measures are implemented to protect patients [16].

Plasma expanders are still widely used for ANH currently. However, existing clinical studies have not focused on large-volume infusion with plasma expanders, and there is a lack of studies that compare the effects of different plasma expanders. Therefore, in the present study, a rodent model of severe ANH was established to compare the effects of different plasma expanders on the blood gas, acid-base balance and physiological parameters. The present findings may provide an experimental basis for large-volume infusion with plasma expanders in clinical practice and will provide an academic reference for the treatment of patients with severe trauma and massive bleeding.

## Methods

### Measurement of physicochemical properties with plasma expanders

Blood samples were collected from Wistar rats. The cone-plate viscometer (BT-300, Bright, China) was used to determine the intrinsic viscosity of the plasma expanders at a shear rate of 200 s^− 1^. Whole blood was centrifuged at 3000 r/min and 4 °C for 10 min, and then the plasma was separated and kept in a centrifuge tube. Red cell concentrates (0.48 ml) were mixed with plasma (0.42 ml) to get red cell suspensions. Plasma or plasma expanders (0.3 ml) were added to the red cell suspensions to get the corresponding mixtures. The mixtures were incubated in a water bath (YHJD-05-1 L, Shanghai Pingxuan Scientific Instrument Co., Ltd., China) at 37 °C for 15 min, and afterward, the viscosity of the mixtures was determined at shear rates of 200, 100, 30, and 1 s^− 1^ using a cone-plate viscometer.

The plasma expanders were diluted with phosphate buffer solution (PBS) to obtain a concentration of 0.05%, and the hydrodynamic radius (R_h_) [17] of each plasma expander was determined by a Zetasizer (Nano2S, Malvern, China) at 25 °C. Plasma expanders in the intrinsic concentration of the injection were diluted with PBS to achieve a concentration of 2%. A colloid osmometer (Osmomat 050, Gonotec, Germany) was used to measure the osmotic pressure of the colloids in the intrinsic concentration and diluted concentration (2%).

### Animals

All experiments were approved by the Laboratory Animal Centre of the Academy of Military Medical Sciences. Since researchers have found that there are gender differences in the morbidity and mortality from trauma and hemorrhagic shock (HS) [18, 19], in order to avoid gender effects and get homogeneous data, 60 male Wistar rats (270–340 g) purchased from Vital River Laboratories (Beijing, China) were randomly divided into five groups in the present study. Each rat was anesthetized via intraperitoneal injection with sodium pentobarbital (Peking Chemical Agent Co., China) (50 mg/kg). The rats were then put in the supine position on a heating pad (TMS-202, Softron, China) with a temperature of 37 °C.

### Experimental animal grouping

The animals were randomly divided into 5 groups (12 rats for each group), as follows: 1) Gel group, 4% Succinylated Gel (Gelofusine®, B. Braun, Shenyang, China); 2) HES200 group, 6% HES 200/0.5 (HAES-steril®, Fresenius-Kabi, Bad Homburg, Germany); 3) HES130 group, 6% HES 130/0.4 (Voluven®, Fresenius-Kabi, Bad Homburg, Germany); 4) HES40 group, 6% HES40 (Shandong Qidu Pharmaceutical Co. China); and 5) Dex40 group, 6% Dextran 40 (Shandong Qidu Pharmaceutical Co. China). Each injection contained NaCl solution (0.9%).

### Rodent model of ANH

Both femoral arteries and the right femoral vein were isolated and cannulated with polyethylene catheters (PE-50). The catheter inserted into the left femoral artery was used for blood withdrawal at the speed of 20 ml/h [20, 21], the catheter inserted in the right femoral vein was used for fluid infusion at the same speed, and the catheter in the right femoral artery was connected to a multiple-channel recorder (MP150, Biopac System, USA) for monitoring the blood pressure and heart rate (HR). The left jugular vein was isolated and cannulated to approximately 3.5 cm deep with a catheter for the measurement of the central venous blood gas.

**Fig. 1:**
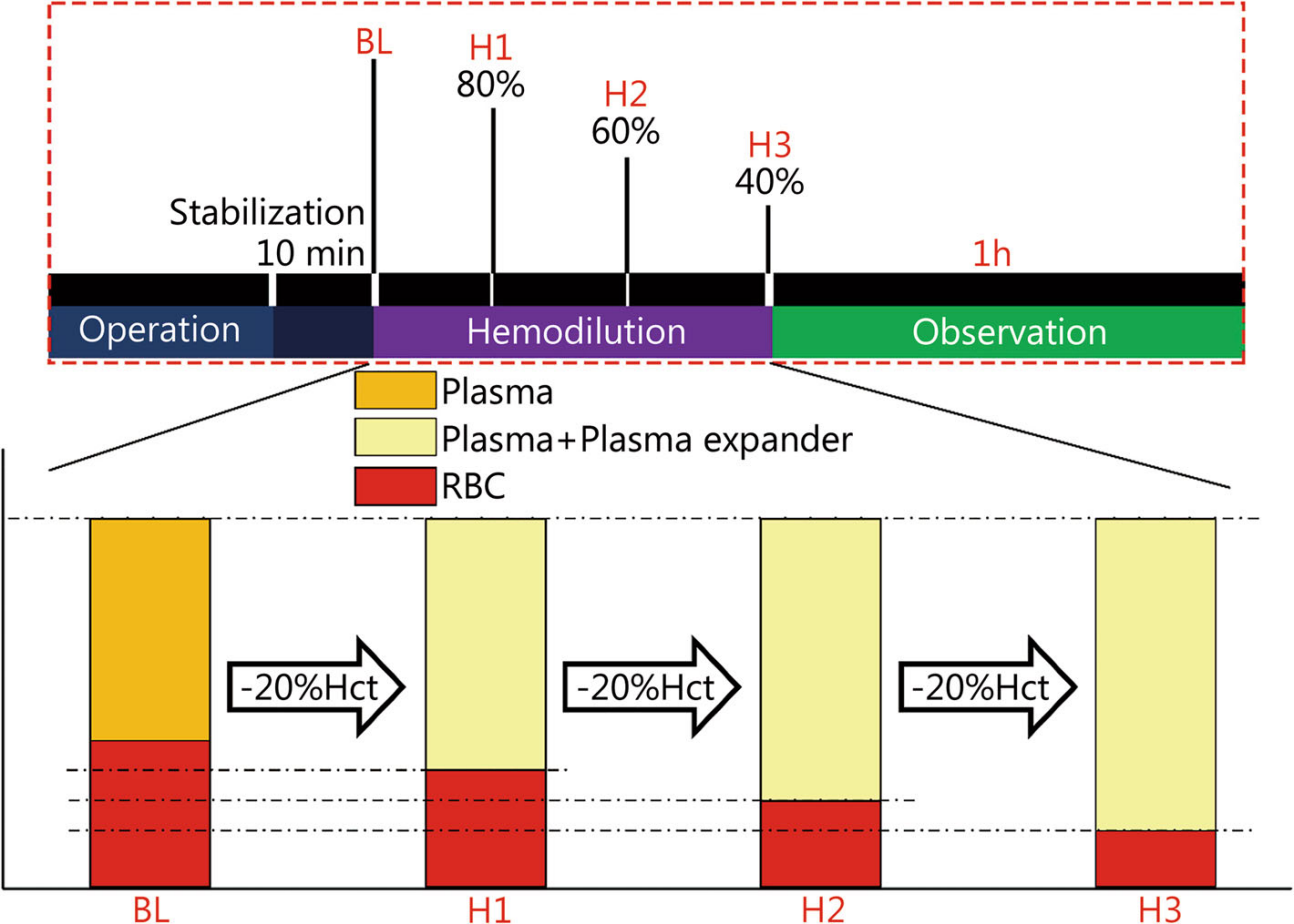
Preparation of ANH. ANH. Acute normovolemic hemodilution; BL. Baseline; H1. The end of the first step of hemodilution; H2. The end of the second step of hemodilution; H3. The end of the final step of hemodilution; RBC. Red blood cell; Hct. Hematocrit

The hemodilution approach is shown in Fig. 1, and it was performed in three steps. The first step of hemodilution (H1) was stopped when the Hct was reduced to 80% of baseline level, and this took approximately 20 min. In the following steps, the hematocrit (Hct) was reduced to 60% (H2) and 40% (H3) of the baseline level, taking approximately 30 min and 45 min, respectively. H3 was the end of ANH. After H3, the rats were monitored for 60 min. The assessment time points were the baseline (BL), the end of the first step of hemodilution (H1), the end of the second step of hemodilution (H2), the end of the final step of hemodilution (H3), 10 min after H3 (10 min), 20 min after H3 (20 min), 30 min after H3 (30 min) and 60 min after H3 (60 min). At the end of the experiment, according to the regulations for the administration of affairs concerning experimental animals, the animals were killed by cervical dislocation under anesthesia.

### Measurement of blood parameters

The Hct and hemoglobin (Hb) concentrations were measured by a semiautomated blood cell analyzer (Hemavet 950, Drew Scientific Inc., USA). The blood gas was determined by a blood gas analyzer (ABL80 FLEX, Radiometer Copenhagen, Denmark).

### Statistical analysis

All data were examined for normality and homogeneity of variance. Comparisons among all groups at a single time point or among all time points within one group were performed using one-way independent ANOVA followed by Fisher’s (LSD) post hoc analysis when the normal distribution or homogeneity of variance assumption was satisfied, and otherwise, the nonparametric Kruskal-Wallis test was used. The correlations between physiological parameters and blood gas parameters were analyzed by multiple linear regression, and the independent variables were screened by a step-by-step method. The correlation between the COP and R_h_ of colloids was analyzed by two individual regression analysis. SAS 9.2 software (SAS Institute Inc., Cary, USA) was used to analyze the data, which are expressed as the mean ± standard deviation (M ± SD). *P* < 0.05 was considered to indicate a statistically significant difference.

## Results

### Physicochemical properties of plasma expanders

**Fig. 2:**
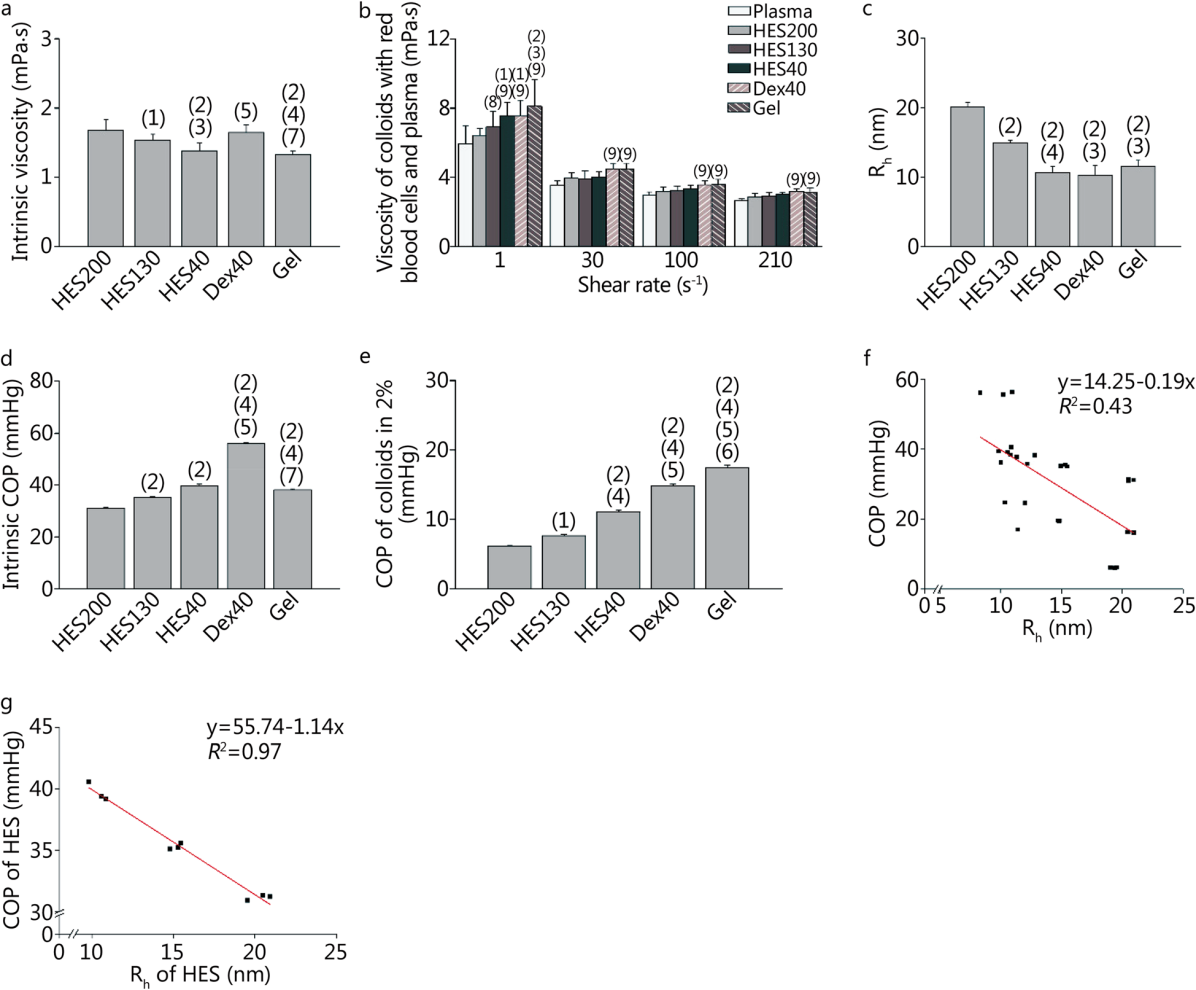
Physicochemical property parameters and the correlation between the COP and R_h_. **a**. Intrinsic viscosity of colloids; **b**. Viscosity of mixtures with plasma or plasma expanders; **c**. R_h_; **d**. The COP of colloids in intrinsic concentration; **e**. The COP of colloids at a diluted concentration (2%); **f**. The correlation between COP and R_h_ of different colloids (*P* < 0.01); **g**. The correlation between COP and R_h_ of HES (*P* < 0.01). COP. Colloid osmotic pressure; R_h_. Hydrodynamic radius. (1) *P* < 0.05, (2) *P* < 0.01 compared with HES200; (3) *P* < 0.05, (4) *P* < 0.01 compared with HES130; (5) *P* < 0.01 compared with HES40; (6) *P* < 0.05, (7) *P* < 0.01 compared with Dex40; (8) *P* < 0.05, (9) *P* < 0.01 compared with Plasma

Figure 2a shows the intrinsic viscosity of the colloids. Gel had a lower intrinsic viscosity than Dex40, HES200, and HES130 (*P <* 0.01). Dex40 had a higher intrinsic viscosity than that of HES40 (*P <* 0.01). The viscosities of HES200, HES130 and HES40 decreased with the decrease of the molecular weight. The viscosities of the different colloids mixed with red cells and plasma at various shear rates are demonstrated in Fig. 2b. At a shear rate of 1 s^− 1^, the mixture containing Gel had a higher viscosity than the mixtures containing plasma, HES200 and HES130 (*P* < 0.05 or *P* < 0.01). The mixtures containing HES40 and Dex40 had higher viscosities than the mixtures containing plasma and HES200 (*P* < 0.05 or *P* < 0.01). The mixture containing HES130 had a higher viscosity than the mixture containing plasma (*P* < 0.05). The mixtures containing Gel and Dex40 had higher viscosities than the mixture containing plasma at 30 s^− 1^, 100 s^− 1^, and 210 s^− 1^ (*P* < 0.01). Furthermore, Fig. 2c demonstrates the R_h_ values of the different plasma expanders. The R_h_ of HES200 was larger than the others (*P* < 0.01). HES130 had a larger R_h_ than HES40 (*P* < 0.01), Dex40 and Gel (*P* < 0.05).

Figure 2d shows the colloid osmotic pressure (COP) of plasma expanders at the intrinsic concentration of the injection, which was 4% for Gel and 6% for the others. The COP of HES200 was significantly lower than those of the other colloids (*P* < 0.01). The COP of Dex40 was significantly higher than those of the other colloids (*P* < 0.01). The COP of Dex40 and Gel were significantly higher than that of HES130 (*P* < 0.01). To measure the COP at the same concentration, the colloids were diluted with PBS to achieve a concentration of 2%, which is shown in Fig. 2e. HES200 had the lowest COP. HES40 had a higher COP than HES130 (*P* < 0.01). Dex40 had a higher COP than HES130 and HES40 (*P* < 0.01). The Gel had the highest COP.

The correlation between R_h_ and COP of all colloids is demonstrated in Fig. 2f, and the coefficient of determination (*R*^2^) was 0.43, which was statistically significant (*P* < 0.01). Furthermore, the correlations between the R_h_ and COP of HES200, HES130 and HES40, which are all hydroxyethyl starches, are shown in Fig. 2g. The coefficient of determination (*R*^2^) was 0.97, which was statistically significant (*P* < 0.01).

### Preparation of ANH model

**Fig. 3:**
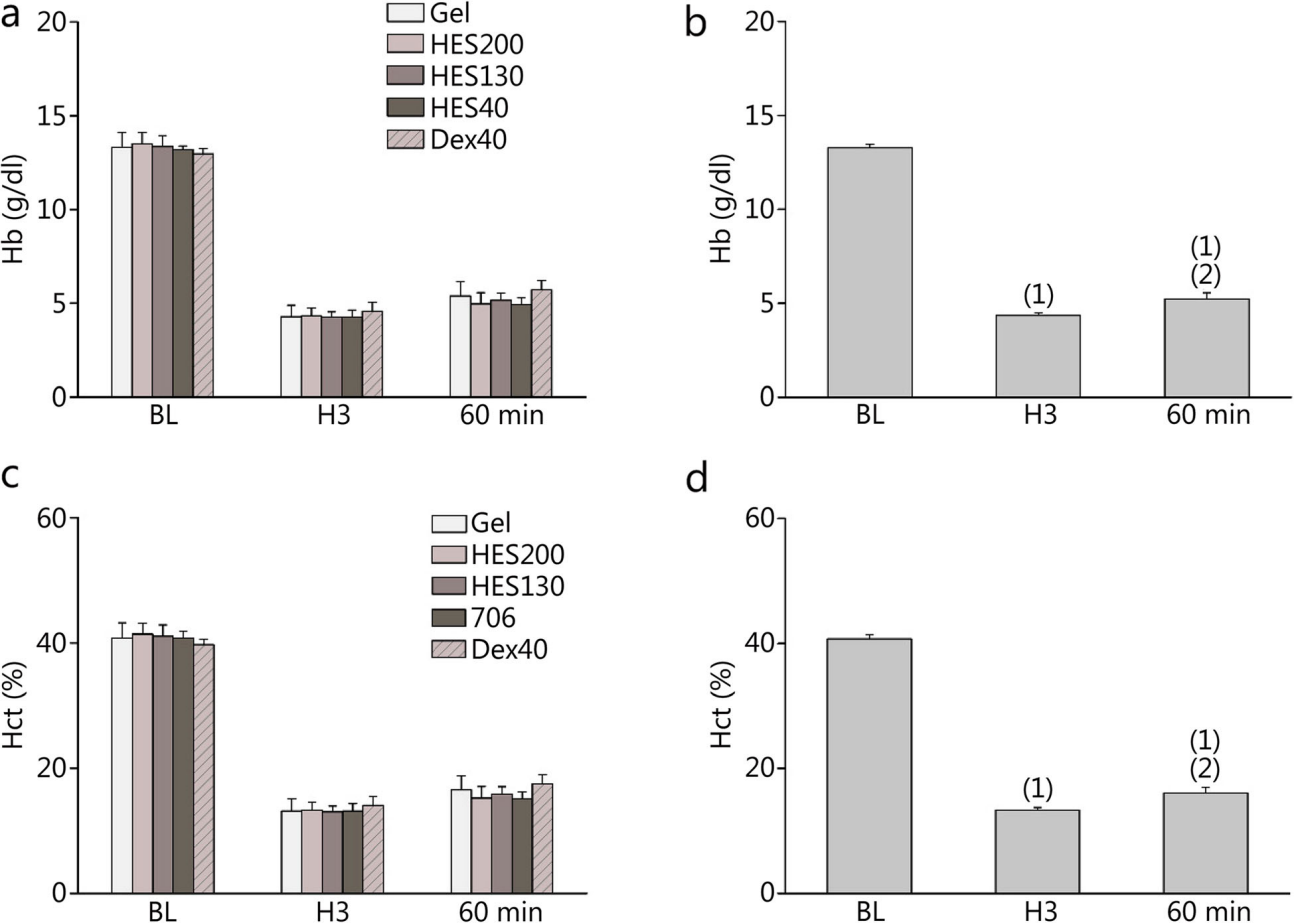
Hematocrit (Hct) and hemoglobin (Hb) of rats that underwent ANH. **a**, **b**. Hb at BL, H3 and 60 min; **c**, **d**. Hct at BL, H3 and 60 min. (1) *P* < 0.01 compared with BL; (2) *P* < 0.01 compared with H3

The hemoglobin (Hb) concentration and Hct are demonstrated in Fig. 3. There were no significant differences between groups at any time point (Fig. 3a, c). Hb and Hct were lower at H3 and 60 min than at BL (*P* < 0.01, Fig. 3b), and they become higher at 60 min than at H3 (Fig. 3d).

### Acid-base and lactate parameters

**Fig. 4:**
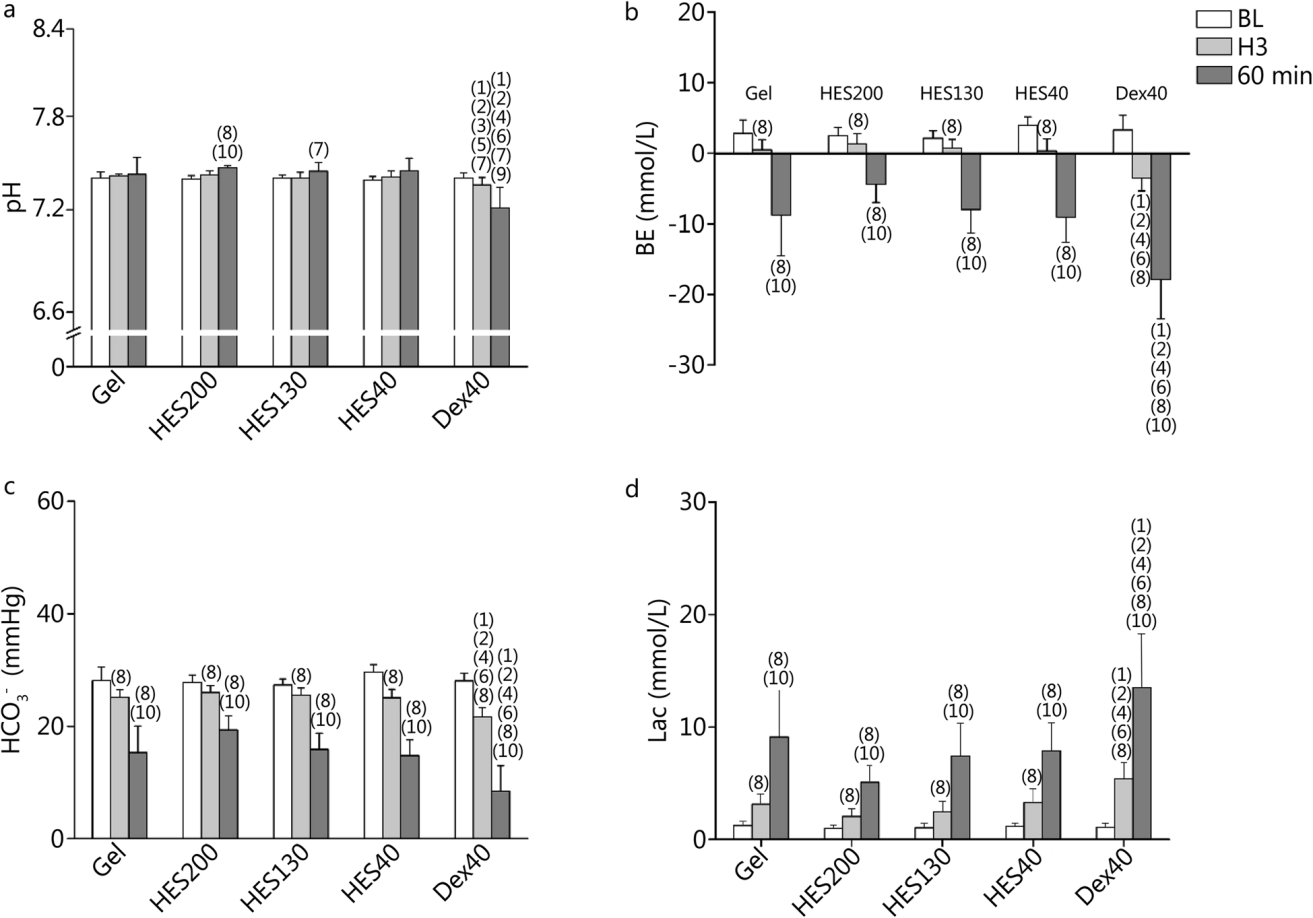
Acid-base and lactate parameters of rats that underwent ANH. **a**. pH at BL, H3 and 60 min; **b**. Base excess (BE) at BL, H3 and 60 min; **c**. Bicarbonate ion (HCO3^−^) at BL, H3 and 60 min; **d**. Blood lactate (Lac) at BL, H3 and 60 min. (1) *P* < 0.01 compared with Gel; (2) *P* < 0.01 compared with HES200; (3) *P* < 0.05, (4) *P* < 0.01 compared with HES130; (5) *P* < 0.05, (6) *P* < 0.01 compared with HES40; (7) *P* < 0.05, (8) *P* < 0.01 compared with BL; (9) *P* < 0.05, (10) *P* < 0.01 compared with H3

The acid-base balance was examined by measuring the pH, base excess (BE), bicarbonate ion concentration (HCO_3_^−^) [22], and blood lactate concentration (Lac) to assess the tissue hypoxia of rats together with the blood gas parameters. The pH in the HES200 group was increased significantly at 60 min compared with BL and H3 (*P* < 0.01, Fig. 4a). In the Dex40 group, the pH decreased gradually at H3 and 60 min compared with BL (*P* < 0.05). The pH at H3 and 60 min was lower in the Dex40 group than in the other groups (*P* < 0.05 or *P* < 0.01).

In all groups, the actual BE levels and the HCO_3_^−^ levels of all groups decreased gradually from BL to 60 min after ANH (*P* < 0.01, Fig. 4b, c). At H3 and 60 min, the BE and HCO_3_^−^ levels were lower in the Dex40 group than in the other groups (*P* < 0.01). The Lac of all groups increased gradually from BL to 60 min (*P* < 0.01, Fig. 4d). The Lac was higher in the Dex40 group than in the other groups at H3 and 60 min (*P* < 0.01).

### Blood gas parameters

**Fig. 5:**
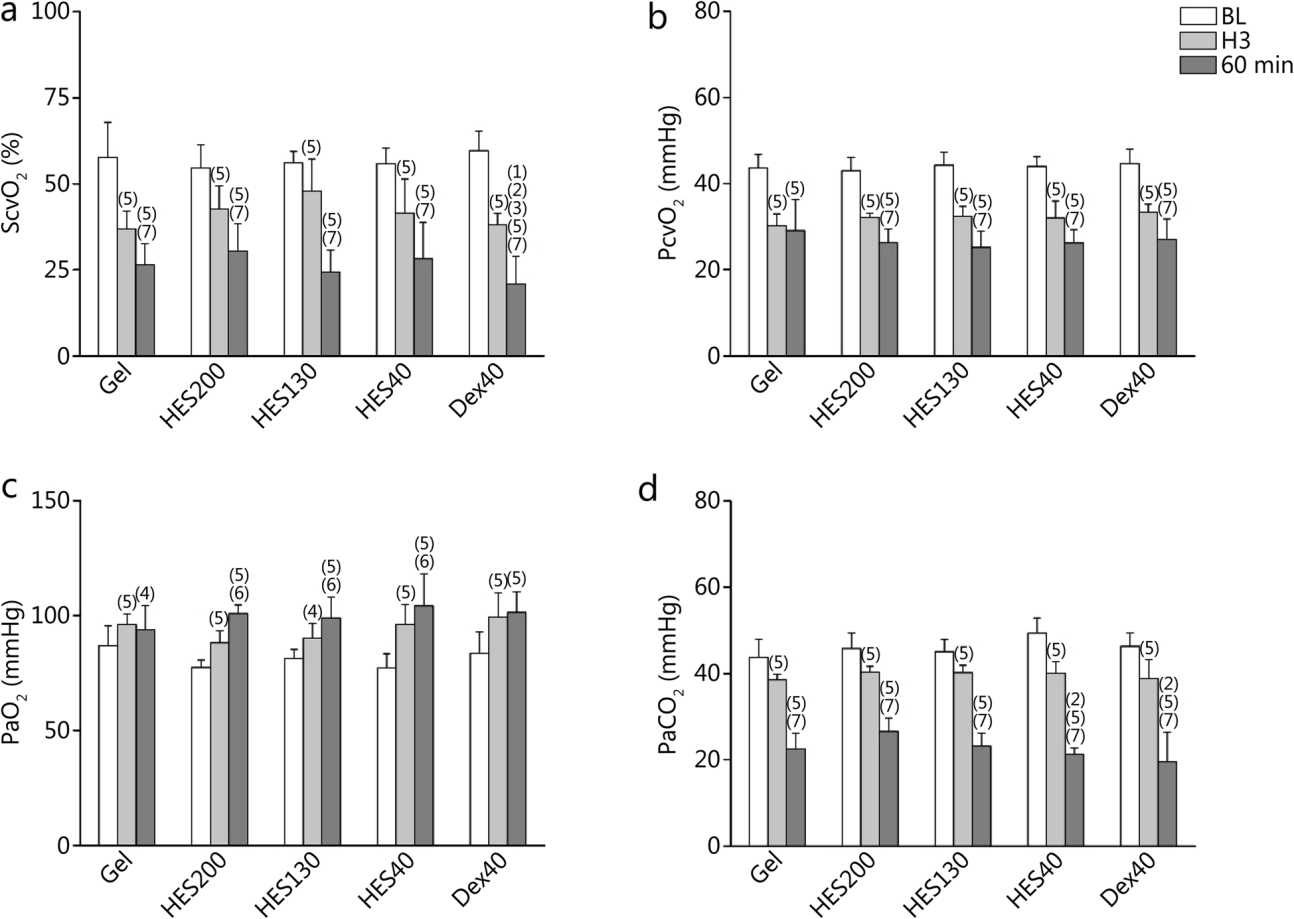
Blood gas parameters of rats that underwent ANH. **a**. Oxygen saturation in central venous blood (ScvO_2_) at BL, H3 and 60 min; **b**. Central venous oxygen partial pressure (PcvO_2_) at BL, H3 and 60 min; **c**. Arterial carbon dioxide partial pressure (PaO_2_) at BL, H3 and 60 min; **d**. Arterial oxygen partial pressure (PaCO_2_) at BL, H3 and 60 min. (1) *P* < 0.05 compared with Gel; (2) *P* < 0.05 compared with HES200; (3) *P* < 0.05 compared with HES40; (4) *P* < 0.05, (5) *P* < 0.01 compared with BL; (6) *P* < 0.05, (7) *P* < 0.01 compared with H3

To assess the tissue hypoxia of rats, the oxygen saturation in the central venous blood (ScvO_2_), central venous oxygen partial pressure (PcvO_2_), partial pressure of oxygen (PaO_2_) and partial pressure of carbon dioxide (PaCO_2_) were measured [23] and are demonstrated in Fig. 5. The ScvO_2_ (Fig. 5a), PcvO_2_ (Fig. 5b) and PaCO_2_ (Fig. 5d) of all groups decreased gradually (*P* < 0.01) and the PaO_2_ (Fig. 5c) increased gradually (*P* < 0.05 or *P* < 0.01) from BL to 60 min after ANH. The ScvO_2_ was lower in the Dex40 group than in the Gel, HES200 and HES40 groups at 60 min (*P* < 0.05). The PaCO_2_ was higher in the HES200 group than in the HES40 and Dex40 groups at 60 min (*P* < 0.05).

### Physiological parameters

To assess the cardiac function, tissue infusion, and microcirculation [24], the systolic blood pressure (SBP), diastolic blood pressure (DBP), mean arterial pressure (MAP), pulse pressure (PP) and heart rate (HR) were monitored. The SBP, DBP and MAP levels were significantly decreased (*P* < 0.01) at H3 compared with BL in all groups. The SBP increased significantly at 60 min in the HES200 and HES40 groups (*P* < 0.05), and the DBP and MAP decreased significantly (*P* < 0.01) at 60 min in the Dex40 group compared with those at H3 (Table 1).

**Table 1:** The physiological parameters of rats that underwent ANH at different times with different plasma expanders ($\overline{x}\pm s$, *n* = 12)

Group	BL	H1	H2	H3	10 min	20 min	30 min	60 min
Systolic blood pressure (SBP)								
Gel	140.168 ± 11.859	136.240 ± 16.364	108.203 ± 14.858	99.217 ± 12.332^(10)^	108.868 ± 16.604	116.213 ± 19.754	122.789 ± 23.135	121.478 ± 32.392^(9)^
HES200	139.123 ± 13.148	138.648 ± 13.217	137.230 ± 13.444^(2)^	110.098 ± 20.619^(10)^	105.164 ± 20.743	125.580 ± 34.151	130.991 ± 32.423	140.127 ± 40.836^(11)^
HES130	138.393 ± 4.859	137.778 ± 16.099	133.551 ± 13.411^(2)^	113.581 ± 17.696^(10)^	127.529 ± 21.751	126.629 ± 27.639	131.209 ± 29.269	130.090 ± 33.383
HES40	141.577 ± 6.409	126.817 ± 13.688	115.416 ± 27.967^(4) (5)^	86.612 ± 17.932^(3) (5) (10)^	93.876 ± 24.222^(6)^	115.765 ± 27.760	131.297 ± 26.220	130.018 ± 35.846^(11)^
Dex40	142.231 ± 14.095	64.673 ± 14.231^(2) (4) (6) (7)^	106.242 ± 20.505^(4) (6)^	103.146 ± 24.677^(10)^	105.697 ± 27.972	109.319 ± 30.351	110.334 ± 32.954	86.488 ± 43.068^(1) (4) (5) (7) (10)^
Diastolic blood pressure (DBP)								
Gel	94.853 ± 8.881	81.576 ± 11.379	55.466 ± 9.690	46.653 ± 8.872^(10)^	46.849 ± 7.914	47.318 ± 8.521	49.323 ± 11.481	43.576 ± 9.762^(10)^
HES200	93.682 ± 12.993	82.916 ± 9.286	74.398 ± 9.178	52.788 ± 10.263^(10)^	48.952 ± 10.530	52.568 ± 11.833	51.080 ± 9.613	54.891 ± 13.559^(10)^
HES130	92.796 ± 6.813	76.879 ± 11.935	65.290 ± 9.624^(1)(3)^	46.919 ± 6.062^(10)^	49.234 ± 6.613	46.686 ± 8.530	50.147 ± 11.068	53.093 ± 14.453^(10)^
HES40	95.000 ± 7.770	75.343 ± 12.010^(4)^	58.860 ± 10.754^(4)^	42.429 ± 9.303^(4)(10)^	39.901 ± 9.207^(3) (5)^	43.588 ± 8.055^(5)^	47.657 ± 4.737	44.543 ± 9.174^(10)^
Dex40	95.284 ± 11.680	38.584 ± 6.161^(2) (4) (6) (8)^	59.405 ± 9.050^(4)^	48.187 ± 6.088^(10)^	41.532 ± 4.152	41.864 ± 8.027^(3)^	39.731 ± 7.165^(1) (3) (5)^	37.158 ± 7.677^(2) (4) (6) (10)^
Mean arterial pressure (MAP)								
Gel	111.752 ± 8.667	100.658 ± 10.970	75.215 ± 11.170	64.753 ± 9.945^(10)^	65.321 ± 8.045	67.178 ± 9.241	68.885 ± 10.102	63.618 ± 14.470^(10)^
HES200	113.651 ± 9.458	106.256 ± 7.667	97.344 ± 9.185^(2)^	74.505 ± 13.152^(1) (10)^	67.913 ± 13.060	73.728 ± 14.269	74.898 ± 10.664	75.712 ± 13.547^(10)^
HES130	109.871 ± 7.333	97.837 ± 9.597	90.348 ± 5.013^(2)^	68.081 ± 5.942^(10)^	71.926 ± 5.203	68.477 ± 9.812	71.109 ± 11.126	70.802 ± 13.674^(10)^
HES40	112.675 ± 6.884	92.496 ± 12.882^(4)^	78.461 ± 15.922^(4) (5)^	59.537 ± 12.138^(4) (10)^	57.265 ± 10.350^(3) (6)^	66.001 ± 6.237	71.123 ± 6.282	66.339 ± 11.590^(4) (10)^
Dex40	115.122 ± 7.716	47.732 ± 7.961^(2) (4) (6) (8)^	76.580 ± 12.221^(3) (6)^	68.285 ± 9.118^(10)^	59.605 ± 5.867^(5)^	63.803 ± 14.947^(3)^	57.754 ± 8.092^(1) (4) (5) (8)^	47.899 ± 15.187^(2) (3) (4) (6) (10) (12)^
Pulse pressure (PP)								
Gel	94.853 ± 8.881	81.576 ± 11.379	55.466 ± 9.690	46.653 ± 8.872^(9)^	46.849 ± 7.914	47.318 ± 8.521	49.323 ± 11.481	43.576 ± 9.762^(10) (11)^
HES200	93.682 ± 12.993	82.916 ± 9.286	74.398 ± 9.178	52.788 ± 10.263^(9)^	48.952 ± 10.530	52.568 ± 11.833	51.080 ± 9.613	54.891 ± 13.559^(9)^
HES130	92.796 ± 6.813	76.879 ± 11.935	65.290 ± 9.624	46.919 ± 6.062^(10)^	49.234 ± 6.613	46.686 ± 8.530	50.147 ± 11.068	53.093 ± 14.453
HES40	95.000 ± 7.770	75.343 ± 12.010	58.860 ± 10.754	42.429 ± 9.303	39.901 ± 9.207^(5)^	43.588 ± 8.055	47.657 ± 4.737	44.543 ± 9.174^(10) (11)^
Dex40	95.284 ± 11.680	38.584 ± 6.161^(2)(4)(6)(8)^	59.405 ± 9.050	48.187 ± 6.088	41.532 ± 4.152	41.864 ± 8.027	39.731 ± 7.165	37.158 ± 7.677^(3)^
Heart rate (HR)								
Gel	420.289 ± 53.954	434.743 ± 52.869	394.134 ± 57.146	410.907 ± 26.481	407.544 ± 5.332	426.433 ± 10.575	444.577 ± 18.442	475.540 ± 24.602^(10) (12)^
HES200	427.301 ± 37.580	418.391 ± 38.261	434.958 ± 32.169	422.450 ± 42.006	384.181 ± 46.764	403.069 ± 41.881	413.515 ± 42.572	444.300 ± 42.493
HES130	385.530 ± 43.569	409.406 ± 40.672	418.329 ± 44.260	420.304 ± 37.465	420.787 ± 35.688	430.079 ± 30.444	415.786 ± 48.098	479.939 ± 53.086^(10) (12)^
HES40	430.198 ± 29.904	424.210 ± 43.051	424.833 ± 36.148	401.392 ± 50.950	408.080 ± 55.381	441.905 ± 58.796	453.675 ± 51.001	486.037 ± 32.176^(10) (12)^
Dex40	392.847 ± 33.640	420.452 ± 63.136	439.580 ± 15.382^(1)^	460.738 ± 28.251^(1)(7)(10)^	471.792 ± 44.495^(2) (4) (5) (8)^	476.878 ± 47.590^(2) (4) (5)^	479.893 ± 45.129^(4) (6)^	475.450 ± 31.951^(10)^

(1) *P* < 0.05, (2) *P* < 0.01 compared with Gel; (3) *P* < 0.05, (4) *P* < 0.01 compared with HES200; (5) *P* < 0.05, (6) *P* < 0.01 compared with HES130; (7) *P* < 0.05, (8) *P* < 0.01 compared with HES40; (9) *P* < 0.05, (10) *P* < 0.01 compared with BL; (11) *P* < 0.05, (12) *P* < 0.01 compared with H3

The SBP, DBP and MAP levels decreased during the ANH. The SBP levels soon recovered towards the normal level during the observation phase, while the DBP and MAP maintained decreased levels during the observation phase. The SBP, DBP and MAP levels decreased rapidly and suddenly in the Dex40 group at H1, with values lower than those in the other groups (*P* < 0.01). Then, it soon recovered at H2 but was significantly lower than in the HES200 and HES130 groups (*P* < 0.05 or *P* < 0.01). The SBP level in the Dex40 group was significantly lower than those in the Gel, HES130, HES40 and HES200 groups at 60 min (*P* < 0.05 or *P* < 0.01). The DBP level in the Dex40 group was significantly lower than that in the HES200 group at H2 and 20 min (*P* < 0.05 or *P* < 0.01), and it was significantly lower than those in the Gel, HES200 and HES130 groups (*P* < 0.05) at 30 min, after which it was significantly lower than those in the HES200 and HES130 groups (*P* < 0.01) at 60 min. The MAP level in the Dex40 group was significantly lower than that in the HES200 group at H2 and those in the other groups at 30 min (*P* < 0.05 or *P* < 0.01), and then it was significantly lower than those in the other groups (*P* < 0.01), except the HES40 group at 60 min.

In addition to the significant differences of the SBP, DBP and MAP between the HES200 group and Dex40 group among time points, the SBP level in the HES200 group was significantly higher than those in the HES40 and Gel groups (*P* < 0.01) at H2, and it was also significantly higher than that in the HES40 group at H1, H3 and 10 min (*P* < 0.05 or *P* < 0.01). Then, the DBP level in the HES200 group was significantly higher than those in the other groups at H2 (*P* < 0.05 or *P* < 0.01), and it was also significantly higher than that in the HES40 group at H3, 10 min and 20 min (*P* < 0.05 or *P* < 0.01). The MAP level in the HES200 group was significantly higher than that in the HES40 group (*P* < 0.01) at H1 and H2, and it was also significantly higher than that in the Gel group at H2, H3 and 60 min (*P* < 0.05 or *P* < 0.01, Table 1).

As displayed in Table 1, the PP level increased significantly at H3 compared with BL in the Gel, HES200 and HES130 groups (*P* < 0.05 or *P* < 0.01), and it increased significantly at 60 min compared with H3 in the Gel and HES40 groups (*P* < 0.05). The HR level significantly increased at 60 min (*P* < 0.01) compared with BL and H3 in the Gel, HES130 and HES40 groups. Furthermore, in the Dex40 group, the HR level increased significantly at H3 and 60 min compared with BL (*P* < 0.01) and was significantly higher than those in the Gel and HES200 groups at H3 (*P* < 0.05, Table 1).

The PP level in the Dex40 group was lower than those in the other groups at H1 (*P* < 0.01). At 10 min, it was higher than that in the HES40 group (*P* < 0.05). At 60 min, it was lower than that in the HES200 group (*P* < 0.05). The HR level in the Dex40 group began to increase significantly from H2. At H2, it was higher than that in the Gel group (*P* < 0.05). At H3, it was higher than those in the Gel and HES40 groups (*P* < 0.05). At 10 min, it was higher than those in the other groups (*P* < 0.05 or *P* < 0.01). At 20 min, it was higher than those in the Gel, HES200 and HES130 groups (*P* < 0.05 or *P* < 0.01). At 30 min, it was higher than those in the HES200 and HES130 groups (*P* < 0.01, Table 1).

**Fig. 6:**
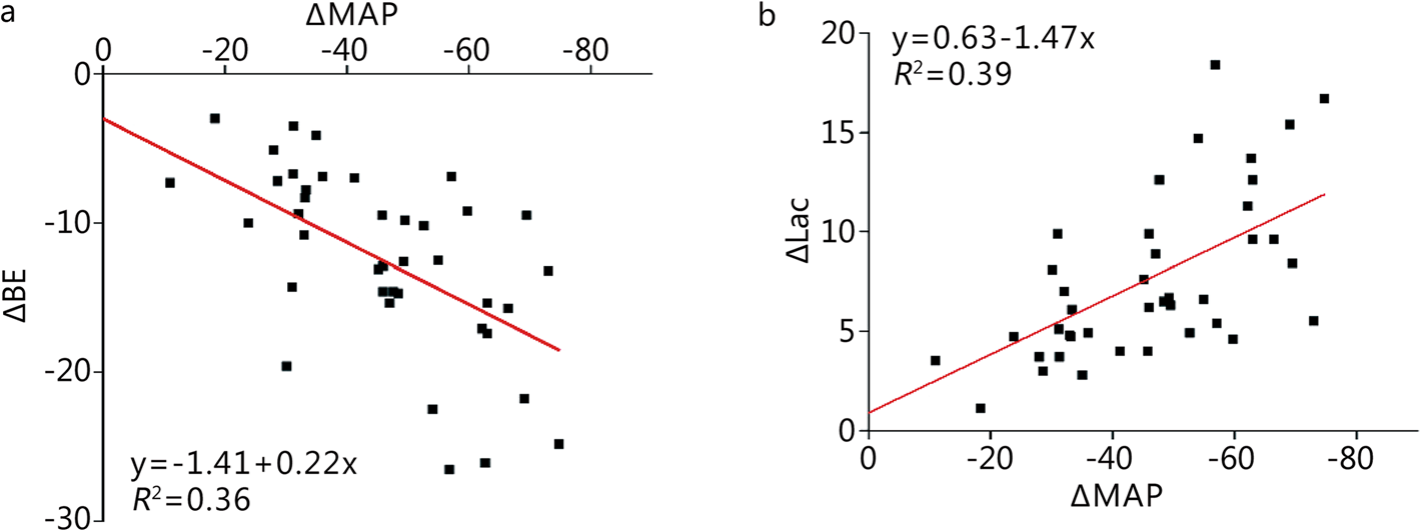
Correlations between physiological parameters and blood gas parameters. **a**. The correlation between △MAP and △BE (*P* < 0.05); **b**. The correlation between △MAP and △Lac (*P* < 0.05)

The correlation between △MAP and △BE is demonstrated in Fig. 6a, and the *R*^2^ was 0.36, which was statistically significant (*P* < 0.05). Furthermore, the correlation between △MAP and △Lac was analyzed and is demonstrated in Fig. 6b. The *R*^2^ was 0.39, which was statistically significant (*P* < 0.05).

## Discussion

### Physicochemical properties of the plasma expanders

In the present study, Gel had a lower intrinsic viscosity than HES200, HES130 and Dex40, but in a mixture with red cells and plasma, Gel had a higher viscosity at low shear rates compared with plasma, HES200 and HES130. Gel also resulted a higher viscosity than plasma at all shear rates. These data are similar to the result of a previous study reporting that Gel increased the blood viscosity [25]. Since viscosity at low shear rates indicates the aggregation of red cells, the results in this study are consistent with the finding that Gel induces red cell aggregation [26].

The R_h_ increased with the increase of the molecular weight. The principle of colloid administration is that solutions containing macromolecules may act as better intravascular volume expanders compared with crystalloid fluids and will increase the plasma COP [9]. For the intrinsic injection concentration, Dex40 had the highest COP, followed by HES40, Gel, HES130, and HES200. This is because the intrinsic concentration of Gel is 4%, while that of the other plasma expanders is 6%. When all colloids were diluted to a concentration of 2%, the COP of Gel was significantly higher than that of Dex40. For all colloids, R_h_ and COP show poor correlation, while for HES40, HES130 and HES200, R_h_ and COP show good correlation. Therefore, at a certain concentration, the COP of hydroxyethyl starch decreases with the increase of the molecular weight.

### The rodent model of severe ANH

The Hct was reduced by approximately 20% in each step (Fig. 1) and was expected to reach 40% of the baseline level at the end of hemodilution. In accordance with accepted standards, the degree of normovolemic hemodilution was classified as mild (Hct > 30%), moderate (Hct 20–30%), or severe (Hct 10–20%) [27]. The present rodent model of ANH reached Hct 16%, indicating severe hemodilution.

At 60 min after H3, the Hct and Hb concentrations were significantly higher than they were at H3, which suggests that the volume expansion effect of the plasma expanders was weakening and that the total circulation volume was reduced because of the in vivo metabolism of the plasma expanders. For example, Dextran can be metabolized into CO_2_ and H_2_O by dextranase at a rate of 70 mg/kg bodyweight every 24 h [28, 29]. The half-life of Dextran (28,000 to 36,000 Da) as determined by its molecular weight in the human body is approximately 30 min [28].

In the preparation of the present rodent model, the speed of infusion or hemorrhage was 20 ml/h, which reduced the Hct to 80, 60, and 40% of the baseline value in the three steps performed to attain ANH (Hct = 16%). Table 2 compares the present rodent model of ANH with the rodent model of HS (hemorrhaging approximately 40% of the total blood volume) which was published in research by our lab [20, 30–32]. In both models, the Hct, Hb, BE, ScvO_2_, and MAP decreased, and the Lac increased. However, the Hct and Hb decreased more in the ANH model than in the HS model. In addition, compared with the HS model, the ANH model resulted in smaller decreases in BE, ScvO_2_, and MAP and a smaller increase in Lac, indicating more severe acidosis in the rodent model of HS [30, 32, 33].

**Table 2:** The differences of variables between the rodent model of ANH and HS

Variables	Severe ANH in this study	HS
Hct (%)	40% of BL	83% of BL [32]
Hb (g/dl)	4.34 ± 0.13	10.70 ± 1.30 [20]
BE (mmol/L)	−0.24 ± 2.23	−13.30 ± 2.70 [30]
MAP (mmHg)	66.86 ± 10.89	38.00–40.00 [30]
Lac (mmol/L)	3.60 ± 1.32	8.60 ± 1.80 [30]
ScvO_2_ (%)	40.13 ± 6.10	35.90 ± 5.60 [20]

*ANH* Acute normovolemic hemodilution, *HS* Hemorrhagic shock, *Hct* Hematocrit, *BL* Baseline, *Hb* Hemoglobin, *BE* Base excess, *MAP* Mean arterial pressure, *Lac* Blood lactate, *ScvO*_*2*_ The oxygen saturation in central venous blood

In the process of model preparation, the SBP levels decreased gradually in all groups and increased back to the level of the baseline except for Dex40, and the DBP and MAP levels in all groups were reduced gradually, which corresponds with previous reporting that human and animal MAP was decreased during the process of ANH [34]. The DBP and MAP levels in all groups were lower than those of the baseline at all time points.

The reason may be that during the hemodilution, although the blood volume did not change significantly, the blood viscosity decreased, so the shear force on the blood vessels decreased, and the tension of the vascular smooth muscle changed, resulting in a decrease in the SBP. In addition, because of the decrease in the blood viscosity, the vascular peripheral resistance decreased, and the diastolic blood flow velocity increased, so the amount of blood retained in the aorta decreased, after which the DBP declined. Moreover, the changes in the MAP, which is approximately equal to the DBP plus 1/3 of the PP, were consistent with the changes in the DBP. The insufficient oxygenation resulted in increased cardiac output due to compensatory mechanisms, so there was an increase in the blood volume ejected into the aorta and the lateral pressure on the arterial wall, which led to an increase in the SBP. In addition, a previous study [35] reported that the HR of patients undergoing ANH was basically unchanged, which was similar to the present findings. However, the HR in the Dex40 group was significantly increased, but the reason for this effect needs further research.

### Influence of different plasma expanders on acid-base, lactate and blood gas parameters

In the present study, the BE was significantly lower in the Dex40 group than in the other groups at H3 and 60 min. The BE, which reportedly reflects the degree of body damage, is markedly decreased in HS [36], demonstrating that the fluctuation of the BE should be closely monitored and can be used to assess the degree of body damage in severe ANH.

At 60 min, the Lac was significantly higher in the Dex40 group than in the other groups. Arterial Lac is a specific product of anaerobic metabolism and could reflect the tissue aerobic metabolism, which is an index of tissue hypoxia [37]. Hyperglycemia reportedly occurs during the early phase of HS [38]. Furthermore, due to the decrease in tissue oxygenation, there is a shift toward anaerobic glycolysis and an increase in Lac [39]. The elimination half-lives of Dex40, HES200, and Gel are 9.6 ± 2.3 h, 12.1 h, and 16.2 h, respectively [28, 40], indicating that Dex40 is metabolized faster than HES200 and Gel. Moreover, at 60 min, the ScvO_2_ was significantly lower in the Dex40 group than in the other groups, which might have been because Dex40 was metabolized rapidly in vivo, resulting in the reduction of its expansion effect, tissue perfusion, and the initiation of tissue hypoxia. ScvO_2_ is an important indicator of the patient’s oxygen delivery, consumption, and cardiac output [41], which are closely connected to tissue hypoxia.

### Influences of different plasma expanders on physiological parameters

The SBP was significantly lower in the Dex40 group than in the other groups at 60 min. The reason for this might be that as the observation period was extended, the Dex40 was rapidly metabolized and excreted in vivo [28]. The concentration of Dex40 in the blood was further decreased because of the hemodilution and metabolism, which reduced the expansion effect of Dex40 and led to a decrease in the blood volume. Thus, the SBP was lower in the Dex40 group than in the other groups and did not return to the baseline level. In addition, the DBP and MAP of the HES200 group were maintained at higher levels compared with the other plasma expanders at some time points.

The SBP, DBP and MAP of anesthetized rats at baseline in this study were approximately 140 mmHg, 94 mmHg and 113 mmHg, respectively. These values are similar to rodent data from the study by Wang *et at* [31]. At H1, nearly 20 min after the beginning of hemodilution, the SBP, DBP and MAP of rats in the Dex40 group decreased to approximately 65 mmHg, 39 mmHg and 47 mmHg, respectively, while those of rats in other groups were approximately 136 mmHg, 79 mmHg and 100 mmHg, respectively. At H2, the SBP, DBP and MAP of rats in the Dex40 group returned to approximately 106 mmHg, 59 mmHg and 77 mmHg, respectively, without significant differences compared with rats in the Gel and HES40 groups. At the same time, rats administered Dex40 didn’t show dyspnea or other symptoms of anaphylactoid reactions [42]; thus, further ANH was continued.

Studies have found that dextran induced hypotension in rats [43, 44]. In a rodent study by Perez-Trepichio et al. [45], intraperitoneal administration of Dex40 before hemodilution and slow infusion of Dex40 during hemodilution avoided peripheral edema and early hypotension. In clinic, dextran-induced anaphylactoid reactions (DIAR) include mild anaphylactoid antibody-independent reactions, and severe anaphylactic reactions [46], with incidences of 1:718 and 1:821, respectively [42]. It seems that in this study, rats administered Dex40 showed mild anaphylactoid antibody-independent reactions, which were approximately of the clinical severity of I or II [42].

Since blood gas measurements are invasive and hard to get in real time, correlation regression analysis between physiological parameters and blood gas parameters was conducted to determine whether physiological parameters can represent changes in blood gas parameters. Regression analysis was conducted on the data of the SBP, DBP, PP, MAP, BE, ScvO_2_ and Lac at BL, H3 and 60 min, as well as the difference value (Δ) between 60 min and BL, in which SBP, DBP, PP and MAP were independent variables, with BE, ScvO_2_ and Lac as dependent variables. Among them, compared with the baseline, changes of the MAP and changes of the BE had coefficients of determination (*R*^2^) of 0.36 and that of MAP and Lac was 0.39, which were the highest two values. Additional studies are needed to find noninvasive measurement methods and build more precise mathematical models since the correlations between physiological parameters and blood gas parameters in the present study are weak.

### Study limitation

The rats in the present study are different from human subjects, so further studies are needed to extend the observation time and clarify the effects of dextran on a normovolemic hemodilution rodent model.

## Conclusions

In the present study, the viscosity, R_h_ and COP of plasma expanders were determined. Gel had a low intrinsic viscosity but may increase the whole-blood viscosity at low shear rates. For hydroxyethyl starch plasma expanders, the COP at a certain concentration decreases with the increase of the R_h_. Namely, the R_h_ and COP showed strong correlation among hydroxyethyl starch plasma expanders. The Hct was reduced to 40% of the baseline level, indicating that the rodent model of severe ANH was built. This change was accompanied by disturbances in the acid-base balance, lactate and blood gas. Dex40 showed a worse outcome in maintaining the acid-base balance and systemic oxygenation than the other plasma expanders. During the process of ANH, Dex40 displayed a V-shaped recovery graph for blood pressure, and the potential mechanism needs further investigation. HES200 had the advantage in sustaining the DBP and MAP at H2 during the ANH. During a 1 h-observation after ANH, Gel, HES200, HES130, HES40 maintained the MAP above 55 mmHg.

## Data Availability

Not applicable.

## Abbreviations

ANH: Acute normovolemic hemodilution
HES: Hydroxyethyl starch
HES200: Hydroxyethyl Starch 200/0.5
HES130: Hydroxyethyl Starch 130/0.4
HES40: Hydroxyethyl Starch 40
Dex40: Dextran 40
Gel: Gelofusine
H1: The end of the first step of hemodilution
H2: The end of the second step of hemodilution
H3: The end of the final step of hemodilution
R_h_: Hydrodynamic radius
COP: Colloid osmotic pressure
Hct: Hematocrit
Hb: Hemoglobin
BE: Base excess
Lac: Blood lactate
HCO_3_^−^: Bicarbonate ion
ScvO_2_: Central venous oxygen saturation
PcvO_2_: Central venous oxygen partial pressure
PaO_2_: Arterial oxygen partial pressure
PaCO_2_: Arterial carbon dioxide partial pressure
HS: Hemorrhage shock
SBP: Systolic blood pressure
DBP: Diastolic blood pressure
MAP: Mean arterial pressure
PP: Pulse pressure
HR: Heart rate
